# Optimizing clinical exome design and parallel gene-testing for recessive genetic conditions in preconception carrier screening: Translational research genomic data from 14,125 exomes

**DOI:** 10.1371/journal.pgen.1008409

**Published:** 2019-10-07

**Authors:** Antonio Capalbo, Roberto Alonso Valero, Jorge Jimenez-Almazan, Pere Mir Pardo, Marco Fabiani, David Jiménez, Carlos Simon, Julio Martin Rodriguez

**Affiliations:** 1 Igenomix Reproductive Genetic Laboratory, Marostica, Italy; 2 DAHFMO Unit of Histology and Medical Embryology, Sapienza University of Rome, Italy; 3 Igenomix, Valencia, Spain; 4 Department of Obstetrics and Gynecology, Valencia University; and INCLIVA, Valencia, Spain; 5 Department of Obstetrics and Gynecology, School of Medicine, Stanford University, Stanford, California, United States of America; Invitae, UNITED STATES

## Abstract

Limited translational genomic research data have been reported on the application of exome sequencing and parallel gene testing for preconception carrier screening (PCS). Here, we present individual-level data from a large PCS program in which exome sequencing was routinely performed on either gamete donors (5,845) or infertile patients (8,280) undergoing in vitro fertilization (IVF) treatment without any known family history of inheritable genetic conditions. Individual-level data on pathogenic variants were used to define conditions for PCS based on criteria for severity, penetrance, inheritance pattern, and age of onset. Fetal risk was defined based on actual carrier frequency data accounting for the specific inheritance pattern (fetal disease risk, FDR). In addition, large-scale application of exome sequencing for PCS allowed a deep investigation of the incidence of medically actionable secondary findings in this population. Exome sequencing achieved remarkable clinical sensitivity for reproductive risk of highly penetrant childhood-onset disorders (1/337 conceptions) through analysis of 114 selected gene-condition pairs. A significant contribution to fetal disease risk was observed for rare (carrier rate < 1:100) and X-linked conditions (16.7% and 41.2% of total FDR, respectively). Subgroup analysis of 776 IVF couples identified 37 at increased reproductive risk (4.8%; 95% CI = 3.4–6.5). Further, two additional couples had increased risk for very rare conditions when both members of a parental pair were treated as a unit and the search was extended to the entire exome. About 2.3% of participants showed at least one pathogenic variant for genes included in the updated American College of Medical Genetics and Genomics v2.0 list of secondary findings. Gamete donors and IVF couples showed similar carrier burden for both carrier screening and secondary findings, indicating no causal relationship to fertility. These translational research data will facilitate development of more effective PCS strategies that maximize clinical sensitivity with minimal counterproductive effects.

## Author summary

We provide here crucial information for optimizing the gene-panel design for preconception carrier screening based on the analysis of a large exome sequencing dataset from infertile individuals and gamete donors. Sequencing the entire coding portion of the human genome combined with separate analysis for few relevant genes offers the possibility to detect most of the pathogenetic variants associated with recessive Mendelian diseases and to develop preconception screening strategies that maximise clinical sensitivity with minimal counterproductive effects. Using a large dataset of individual-level exome sequencing data, we have defined gene specific and aggregate fetal risk detectable for conditions selected on discrete criteria of severity, penetrance, inheritance pattern, and age of onset. About 1 out of 300 affected pregnancies can be detected based on a gene-panel of 114 conditions and ~5% of the couples analysed showed an increased risk that warrant consideration from a reproductive viewpoint. These results suggest the use of exome sequencing and parallel gene testing is clinically effective and feasible for preconception carrier screening after proper validation and translational research has been carried out. However, further studies are necessary to define the best framework for clinical implementation and the actual detection rate of at risk couples.

## Introduction

Emerging evidence shows several advantages of expanding clinical sensitivity to Mendelian recessive diseases in genetic screening of prospective parents (Preconception carrier screening, PCS). Notably, population-based incorporation of parallel screening for cystic fibrosis [CF (MIM: 219700)], fragile X syndrome [FXS (MIM: 300624)], and spinal muscular atrophy [SMA (MIM: 253300)] in routine preconception and early pregnancy programs results in a combined affected pregnancy risk comparable to the risk for Down syndrome[1]. In populations with diverse ethnic backgrounds, expanded carrier screening (ECS) for 94 or 176 severe conditions can significantly increase detection of carrier status compared with current recommendations from professional societies[2,3]. These data further suggest that guidelines recommended by the American College of Obstetricians and Gynaecologists (ACOG) and American College of Medical Genetics and Genomics (ACMG) do not perform equally across racial/ethnic groups, resulting in diverging residual risks and disproportional diagnostic performance. Recently, the scientific societies most actively involved in PCS suggested extending preconception genetic screening to healthy individuals for the most common and most severe recessive conditions[4].

Currently, the debate on ECS is focused on which conditions should be included in the panels and what testing and variant reporting strategy is optimal to maximize clinical sensitivity, cost-effectiveness, and informative value of screening results while minimizing counterproductive effects[5–7]. While recent professional recommendations addressing ECS panel composition offer valuable guidance on test development, most laboratories have established an a priori list of genes and conditions to be tested and disclosed^8^. Arguably, some of these conditions have questionable clinical utility as a result of very low or undetermined carrier frequency, low or unknown testing sensitivity, and mild or incompletely penetrant phenotypes. For this reason, characteristics of tested conditions and the scope of ECS itself should be carefully considered to establish a rational benchmark for providers and patients.

To aid ECS test development, here we report data from a large PCS program in which ECS has been routinely carried out by exome sequencing (ES) in 14,125 gamete donors and couples undergoing IVF without known family history of genetic diseases at the preconception stage. This large individual-level data from ES combined with any a priori selection of conditions to be tested allowed calculation of actual fetal genetic risk at both gene-disease pair and aggregate levels, facilitating the development of an effective gene panel based on clinical validity and actual pathogenic variant frequency data.

In addition, the large-scale application of ES in this study enabled deep investigation of the incidence of medically actionable secondary findings (SF) in the context of PCS. Indeed, as recently recommended by ACMG[8], clinical diagnostic laboratories performing exome or genome sequencing should provide patients with the option to receive information on the pathogenic variants in 59 genes suggested by ACMG SF v2.0, even when unrelated to the primary medical reason for testing.

We show that the use of individual-level translational genomic research data is extremely useful to define an effective PCS strategy able to capture the vast majority of fetal disease risk (FDR) for severe early onset and highly penetrant recessive conditions.

## Results

### Overview of sequencing performance and variants detection

**Fig 1:**
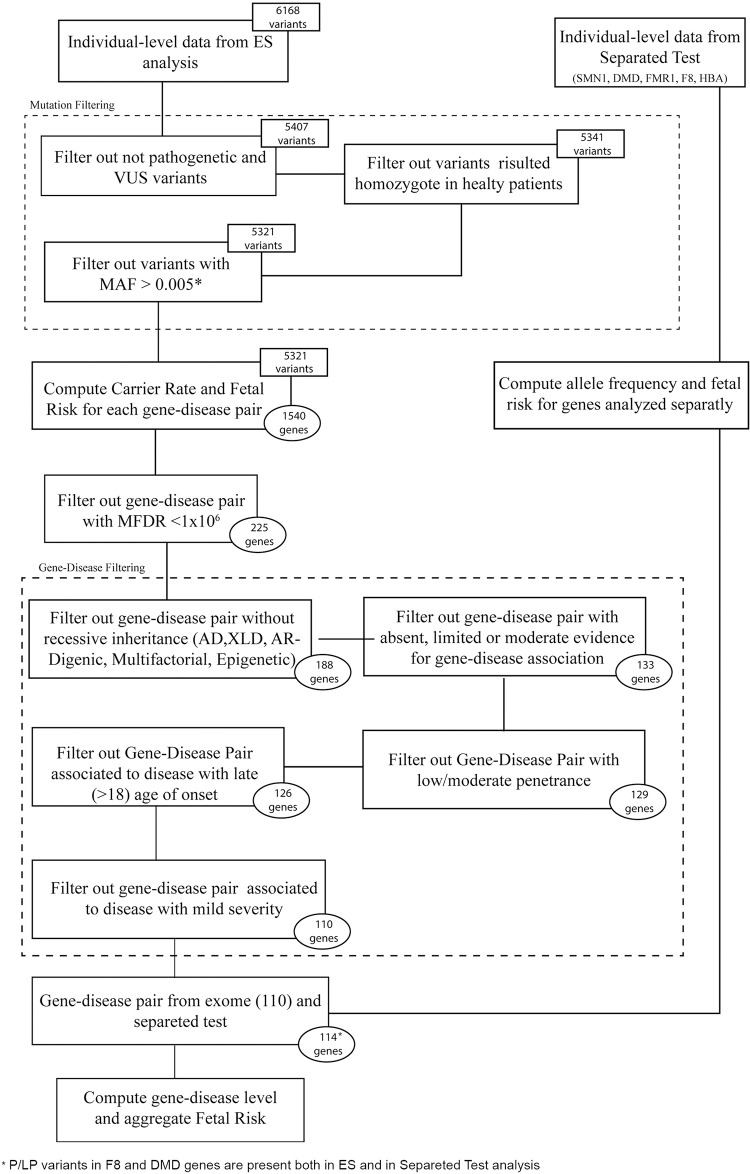
Variant and gene-disease pair selection flowchart.

The depth of coverage was high across exome content, with 94.3% of target regions covered at a depth of at least 30X. Sequences with <10X coverage, ≤20Q and ≤35% heterozygous ratio were not considered for analysis. There were 213 (213/4,814; 4.4%) genes with <90% of the coding sequence inadequately covered by exome sequencing. These genes were still considered for FDR calculation considering the possibility that highly frequent P/LP variants occurring in the well-covered portion of these genes could be clinically relevant. In total, 6,168 SNVs were detected in the clinical exome dataset with a P/LP classification. After variant filtering steps, 5,321 P/LP variants were used to compute carrier rates and FDR for each specific gene-condition pair (Fig 1; S1 Table).

### Gene-disease pair selection

After variant filtering, carrier rate and FDR were calculated by aggregating frequencies of all P/LP variants for each gene and considering the inheritance pattern. The threshold for FDR was set at a disease prevalence of 1 in a million, resulting in a reduction from 1,540 to 225 gene-disease pairs included for further curation (Fig 1; S2 Table). As expected, during this step some gene-disease pairs considered by ACOG[4] as reasonable for inclusion in ECS were excluded [such as familial dysautonomia (MIM: 223900), Fanconi anemia C (MIM: 227645), Joubert syndrome (MIM: 213300), and Bloom syndrome (MIM: 210900)] due to very low carrier rates in our tested population (S2 Table). These conditions are indeed highly prevalent in specific populations and ancestries, such as Ashkenazi, but are usually very rare in Caucasians. Although these conditions were excluded from our gene-disease panel, it is worth noting that they can be relevant for other clinical settings/locations and included in the development of universal ECS.

The remaining 225 conditions were further curated and classified. First, gene-disease pairs with an inheritance pattern other than autosomal or X-linked recessive were removed. A total of 37 gene-disease pairs were filtered out, mainly for association with AD inheritance (S2 Table). Among the most frequently mutated AD genes were germline pathogenic variants for conditions with variable expressivity and mild phenotypes (S2 Table), such as VWF (MIM:613160) involved in von Willebrand disease type 1, FLG (MIM:135940) involved in Ichthyosis vulgaris, PER3 (MIM:603427) involved in Advanced sleep phase syndrome type 3, as well as the cancer predisposition gene RANSEL involved in Prostate cancer 1 (MIM: 180435).

The next filtering step involved conditions with an absent or low/moderate gene-disease association. In this phase, 55 genes were excluded. Next, penetrance was ascertained where possible, and “low” and “mild” penetrance gene-disease pairs were excluded. As expected, *SERPINA1* (MIM: 613490) was the most commonly mutated gene in this category.

Three gene-condition pairs associated with late-onset clinical manifestation were detected and excluded from the carrier list. Some of these were associated with AR cancer, such as *MUTYH* (MIM: 604933), a well-known DNA repair gene in which mutation causes an AR form of familial adenomatous polyposis (MIM: 132600). Finally, classification of severity was consistently applied, and 16 mild conditions were removed from the final list. Including separate gene tests, 114 conditions were available to assess FDR and couple risk (Fig 1).

### FDR according to prevalence and severity classification

**Fig 2:**
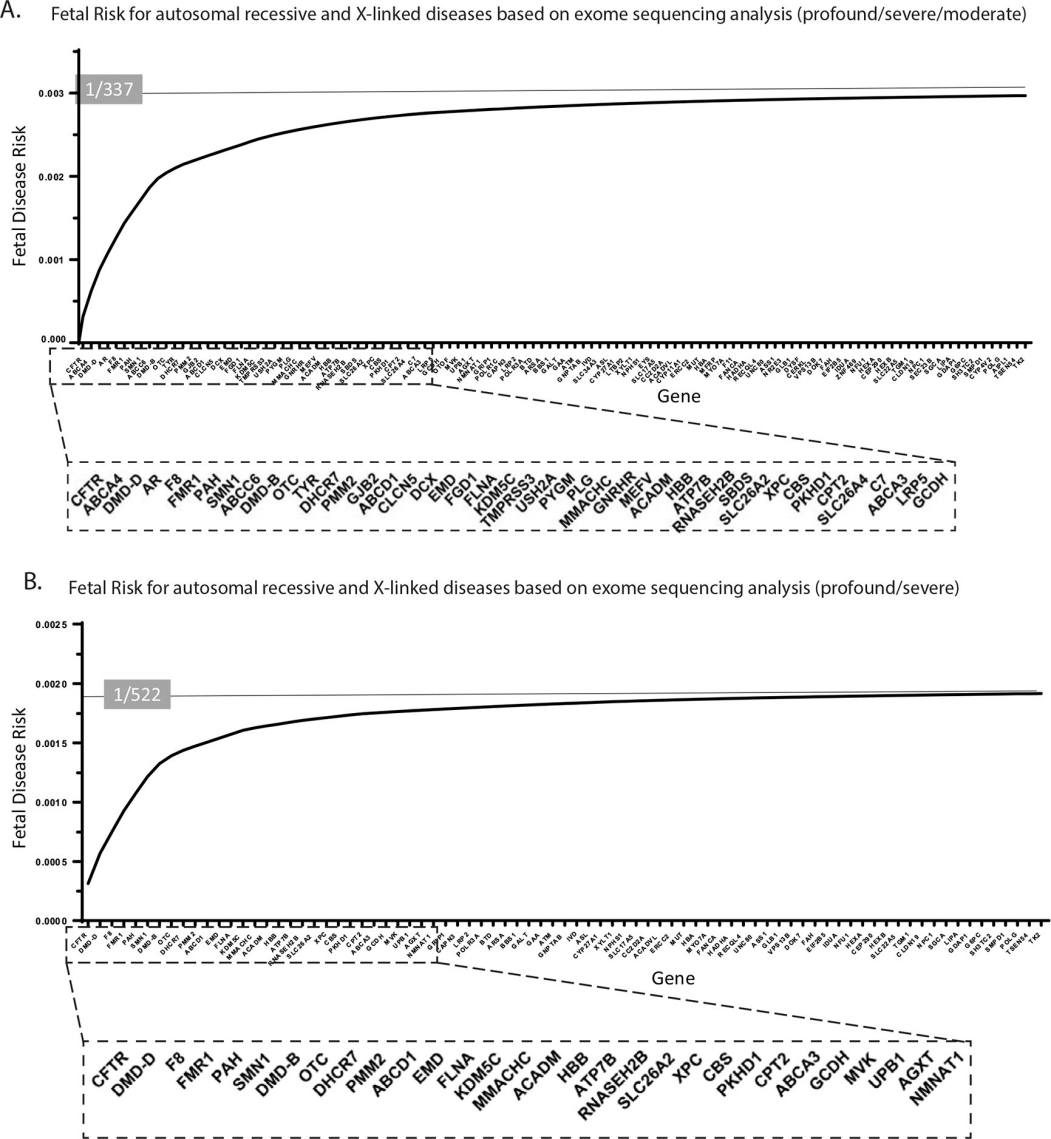
Aggregate fetal disease risk and utility score for 114 conditions from exome sequencing and tests. The full list of genes and the related carrier rate and characteristics are displayed in S2 Table. a) Aggregate fetal disease risk considering only severe and profound conditions. b) Aggregate fetal disease risk with moderate conditions included.

The next steps in our ECS panel-design framework involved definition and representation of gene-level and aggregate clinical sensitivity toward FDR (Fig 2). When considering moderate, severe and profound conditions, aggregated sensitivity toward fetal recessive genetic disease resulted in a predicted rate of 1/337 affected pregnancies (Fig 2A). X-linked and conditions with a carrier rate lower than 1/100 represented 41.2% and 16.7% of the total fetal risk in this analysis, respectively. Moderate conditions alone explained the 35.5% of affected foetuses risk in addition to “severe” and “profound” condition diagnoses.

Aggregated FDR reaches a detection rate of 1/522 affected pregnancy when considering severe and profound conditions only (Fig 2B). A few well-known, highly prevalent severe diseases contribute substantially to overall disease risk in our tested population. In particular, *CFTR* [CF (MIM: 219700), 16.35% FDR], *DMD* [Duchenne muscular dystrophy (MIM: 310200), 13.26% FDR], *F8* [Hemophilia A (MIM: 306700), 9.51% FDR], *FMR1* [FXS (MIM: 300624), 9.21% FDR], *PAH* [Phenylketonuria (MIM: 612349), 7.70% FDR], and *SMN1* [SMA (MIM: 253300), 7.38% FDR] were the top 6 genes providing the highest fetal risk and accounting for ~1 in 823 affected pregnancies and ~60% of overall fetal risk. Further, several of these large contributors, such as *SMN1*, *FMR1*, *F8*, and *DMD*, arise from genes requiring special genetic analysis. X-linked conditions contributed significantly to reproductive risk for severe/profound conditions, representing 39.4% of total FDR. Genes with a carrier rate lower than 1/100 explained 16% of the total FDR for severe/profound conditions. These data highlight that the defined threshold from ACOG guidelines[4] (carrier rate > 1/100 in at least one well-studied population) can result in suboptimal clinical sensitivity, missing a risk of about 1:3000 affected pregnancies in our population.

### Carrier burden for recessive highly penetrant childhood-onset disorders and couple analysis

**Fig 3:**
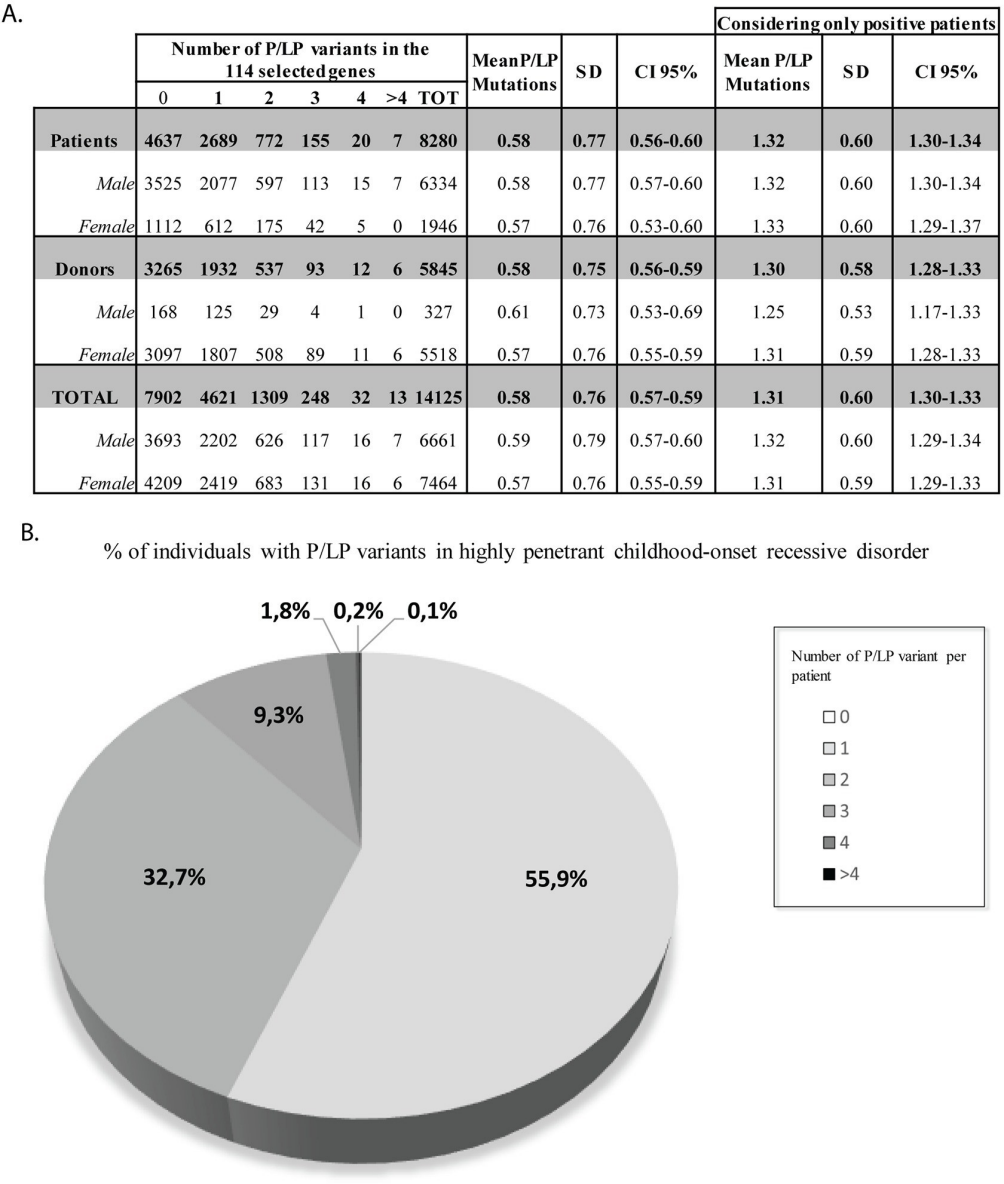
Carrier burden for autosomal and X-linked recessive highly penetrant childhood-onset disorders in the tested population. Data include exome sequencing data from 114 included conditions and separate tests for specific genes. A) Carrier rate metrics for pathogenic (P) and likely pathogenic (LP) variants detected in the cohort of male and female IVF donors and infertile patients. B) Distribution of the number of P/LP variants detected per individual sample.

Among the 14,125 participant samples analysed, 44.1% showed at least one positive carrier result for the 114 selected conditions for ECS. The average number of P/LP variants was 0.58 per individual, with a range of 0–7 variants (1.31 per sample for positive cases; Fig 3A). Donors and patients as well as males and females showed similar carrier burden, suggesting that recessive conditions causing severe and early-onset diseases are not related to fertility.

Subgroup analysis of 776 IVF couples revealed that 37 couples were at increased risk (4.8%; 95% CI = 3.4–6.5) for one of the 114 included conditions (Table 1). Excluding 8 cases with low risk for CGG triplet expansion to the full mutation range in *FMR1* gene, 29 couples were at significantly higher risk (3.7%; 95% CI = 2.5–5.3). Further, 2 additional couples had an increased risk for very rare conditions beyond prevalence of 1 in a million [adenylosuccinate lyase deficiency (MIM: 103050) and microcephaly, epilepsy, and diabetes syndrome (MIM: 614231)] when both parents were treated as a unit and the search for reproductive risk was extended to the entire exome (Table 1). The analysis of commercially available ECS layouts on our couple dataset revealed that some conditions were consistently missing across all the gene-panels (S4 Table), including very frequent moderate (e.g. Stargardt disease, ABCA4; MIM:601691) as well as severe conditions (e.g Hemophilia A, F8; MIM: 300841 and Aicardi-Goutieres syndrome, RNASEH2B; MIM: 610326). This observation might help to further improve and homogenize the global ECS offer.

**Table 1:** Couple at increased risk for autosomal and X-linked recessive conditions.

Partner 1 Pathogenic Variant	Variant Classification	Partner 2 Pathogenic Variant	Variant Classification	Gene	Pathology	Severity	MIM phenotype	MIM gene	CR	FDR
***Conditions with disease prevalence >1 in a milion***										
NM_000350.2:c.634C>T	Pathogenic	NM_000350.2:c.5882G>A	Path/Lik. Path	ABCA4	Stargardt disease	Moderate	604116	601691	1/ 28	0.000310758
NM_000487.5:c.1223_1231delGTGATACCA	Likely path.	NM_000487.5:c.763G>A	Path/Lik. Path	ARSA	Metachromatic leukodystrophy	Profound	250100	607574	1/ 232	4.66254E-06
NM_000492.3:c.3196C>T	Pathogenic	NM_000492.3:c.3196C>T	Pathogenic	CFTR	Cystic fibrosis	Severe	219700	602421	1/ 28	0.000313259
NM_000492.3:c.3846G>A	Pathogenic	NM_000492.3:c.3484C>T	Pathogenic	CFTR	Cystic fibrosis	Severe	219700	602421	1/ 28	0.000313259
NM_000492.3:c.2657+5G>A	Pathogenic	NM_000492.3:c.1040G>A	Pathogenic	CFTR	Cystic fibrosis	Severe	219700	602421	1/ 28	0.000313259
NM_000492.3:c.1624G>T	Pathogenic	NM_000492.3:c.1521_1523delCTT	Pathogenic	CFTR	Cystic fibrosis	Severe	219700	602421	1/ 28	0.000313259
NM_000492.3:c.1647T>G	Pathogenic	NM_000492.3:c.1647T>G	Pathogenic	CFTR	Cystic fibrosis	Severe	219700	602421	1/ 28	0.000313259
NM_000151.3:c.1039C>T	Pathogenic	NM_000151.3:c.1039C>T	Pathogenic	G6PC	Glycogen storage disease Ia	Severe	232200	613742	1/ 441	1.28311E-06
NM_004004.5:c.101T>G	Likely path.	NM_004004.5:c.269T>C	Pathogenic	GJB2	Deafness	Moderate	220290	121011	1/ 85	3.45286E-05
NM_000372.4:c.325G>A	Likely path.	NM_000372.4:c.1217C>T	Path/Lik. Path	TYR	Albinism, oculocutaneous 1	Moderate	203100	606933	1/ 67	5.63164E-05
NM_024312.4:c.3503_3504delTC	Pathogenic	NM_024312.4:c.3503_3504delTC	Pathogenic	GNPTAB	Mucolipidosis II	Profound	252500	607840	1/ 252	3.92952E-06
NM_021150.3:c.160G>A	Likely path.	NM_021150.3:c.160G>A	Likely path.	GRIP1	Fraser syndrome 3	Severe	617667	604597	1/ 221	5.13243E-06
NM_000518.4:c.20A>T	Pathogenic	NM_000518.4:c.20A>T	Pathogenic	HBB	Beta-thalassemia	Severe	613985	141900	1/ 125	0.000016
NM_000518.4:c.27dupG	Pathogenic	NM_000518.4:c.27dupG	Pathogenic	HBB	Beta-thalassemia	Severe	613985	141900	1/ 125	0.000016
NM_001002755.2:c.622G>T	Pathogenic	NM_001002755.2:c.622G>T	Pathogenic	NFU1	Multiple mitochondrial dysfunctions syndrome 1	Profound	605711	608100	1/ 382	1.7154E-06
NM_000277.1:c.1066-11G>A	Pathogenic	NM_000277.1:c.1066-11G>A	Pathogenic	PAH	Phenylketonuria	Severe	261600	612349	1/ 41	0.000147418
NM_024570.3:c.529G>A	Path/Lik. Path	NM_024570.3:c.529G>A	Path/Lik. Path	RNASEH2B	Aicardi-Goutieres syndrome	Severe	610181	610326	1/ 131	1.46154E-05
NM_016038.2:c.258+2T>C	Pathogenic	NM_016038.2:c.258+2T>C	Pathogenic	SBDS	Shwachman-Bodian-Diamond syndrome	Moderate	260400	607444	1/ 135	1.38147E-05
NM_012434.4:c.533delC	Pathogenic	NM_012434.4:c.1138_1139delGT	Path/Lik. Path	SLC17A5	Sialic acid storage disorder, infantile	Profound	269920	604322	1/ 288	3.00854E-06
del ex7-8	Pathogenic	del ex7-8	Pathogenic	SMN1	Spinal muscular atrophy 1	Severe	253300	600354	1/ 42	0.00014144
del ex7-8	Pathogenic	del ex7-8	Pathogenic	SMN1	Spinal muscular atrophy 1	Severe	253300	600354	1/ 42	0.00014144
del ex7-8	Pathogenic	del ex7-8	Pathogenic	SMN1	Spinal muscular atrophy 1	Severe	253300	600354	1/ 42	0.00014144
del ex7-8	Pathogenic	del ex7-8	Pathogenic	SMN1	Spinal muscular atrophy 1	Severe	253300	600354	1/ 42	0.00014144
***Common XLR conditions***										
NM_000531.5:c.422G>A	Pathogenic	/	/	OTC	Ornithine transcarbamylase deficiency	Severe	311250	300461	1/ 3732	6.69882E-05
NM_000044.3:c.2191G>A	Pathogenic	/	/	AR	Androgen insensitivity/Spinal and bulbar muscular atrophy of Kennedy	Moderate	300068	313700	1/ 1244	0.000200965
Inv22	Pathogenic	/	/	F8	Hemophilia A	Severe	306700	300841	1/ 1372	0.000182193
Inv22	Pathogenic	/	/	F8	Hemophilia A	Severe	306700	300841	1/ 1372	0.000182193
Ins Duchenne	Pathogenic	/	/	DMD	Duchenne muscular dystrophy	Severe	310200	300377	1 / 984	0.000253975
Del Duchenne	Pathogenic	/	/	DMD	Duchenne muscular dystrophy	Severe	310200	300377	1 / 984	0.000253975
32.56*	Premutated	/	/	FMR1	Fragile-X Syndrome	Severe	300624	309550	1 /146	0.000176459
22.57*	Premutated	/	/	FMR1	Fragile-X Syndrome	Severe	300624	309550	1 /146	0.000176459
32.63*	Premutated	/	/	FMR1	Fragile-X Syndrome	Severe	300624	309550	1 /146	0.000176459
43.59*	Premutated	/	/	FMR1	Fragile-X Syndrome	Severe	300624	309550	1 /146	0.000176459
24.58*	Premutated	/	/	FMR1	Fragile-X Syndrome	Severe	300624	309550	1 /146	0.000176459
32.57*	Premutated	/	/	FMR1	Fragile-X Syndrome	Severe	300624	309550	1 /146	0.000176459
29.57*	Premutated	/	/	FMR1	Fragile-X Syndrome	Severe	300624	309550	1 /145	0.000176459
28.67*	Premutated	/		FMR1	Fragile-X Syndrome	Severe	300624	309550	1 /145	0.000176459
***Conditions with disease prevalence < 1 in a milion***										
NM_016097.4:c.233T>C	Pathogenic	NM_016097.4:c.233T>C	Pathogenic	IER3IP1	Microcephaly, epilepsy, and diabetes syndrome	Severe	614231	609382	<1 /500	< 0.0000016
NM_000026.3:c.1277G>A	Pathogenic	NM_000026.3:c.1277G>A	Pathogenic	ADSL	Adenylosuccinase deficiency	Severe	103050	608222	<1 /500	< 0.0000016

### Medically actionable SF

Considering only the updated ACMG v2.0 list for SF (59 genes), 218 unique variants occurring a total of 332 times were identified as P/LP (S3 Table). Among the 218 unique putative disease-causing variants, 216 were in AD loci and 2 were in X-linked loci (Table 2). The proportion of participants with at least one P/LP variant in one SF-associated gene was 2.3% (Table 2). Pathogenic variants in *BRCA2* [hereditary breast cancer (MIM: 600185)], *KCNH2* [Romano-Ward long-QT syndrome types 1, 2,and 3, Brugada syndrome (MIM: 600185)], MYBPC3 [Hypertrophic cardiomyopathy, dilated cardiomyopathy (MIM:600958)], LDLR Familial hypercholesterolemia (MIM: 143890)] and RYR1 [Malignant hyperthermia susceptibility (MIM: 145600)]were most prevalent, with a carrier rate of less than 1:700. In addition, 5 individuals had >1 pathogenic variant, and no differences were observed between males and females or between IVF couples and gamete donors regarding carrier burden for SF-associated genes. No homozygous or compound heterozygous individuals for *ATP7B* (MIM: 606882) or *MUTYH* (MIM: 604933) were identified in this cohort.

**Table 2:** Detection of secondary findings from exome sequencing of 14,125 individuals.

Phenotype	OMIM disorder	Typical age of onset	Gene	OMIM gene	Inheritance	CR	Patient Female	Donor Female	Patient Male	Donor Male
Hereditary breast and ovarian cancer	604370	Adult	BRCA1	113705	AD	1/ 706	2	5	11	2
	612555		BRCA2	600185	AD	1/ 248	7	19	28	3
Li-Fraumeni syndrome	151623	Child/adult	TP53	191170	AD	1/ 2825	2	1	1	1
Familial adenomatous polyposis	175100	Child/adult	APC	611731	AD	1/ 14125	0	1	0	0
Lynch syndrome	158320	Adult	MLH1	120436	AD	1/ 3531	0	1	3	0
	120435	Adult	MSH2	609309	AD	1/ 2354	0	3	3	0
	614350	Adult	MSH6	600678	AD	1/ 1284	1	6	4	0
	614337	Adult	PMS2	600259	AD	1/ 1009	4	5	5	0
Von Hippel–Lindau syndrome	193300	Child/adult	VHL	608537	AD	1/ 7063	1	1	0	0
Tuberous sclerosis complex	191100	Child	TSC1	605284	AD	1/ 2018	3	1	3	0
Retinoblastoma	180200	Child	RB1	614041	AD	1/ 14125	0	0	1	0
Hereditary paraganglioma-pheochromocytoma syndrome	168000 (PGL1)	Child/adult	SDHD	602690	AD	1/ 14125	1	0	0	0
	605373 (PGL3)		SDHC	602413	AD	1/ 3531	0	2	2	0
	115310 (PGL4)		SDHB	185470	AD	1/ 4708	1	0	2	0
Marfan syndrome, Loeys-Dietz syndromes,and familial thoracic aortic aneurysms and dissections	154700		FBN1	134797	AD	1/ 942	2	7	5	1
	609192	Child/adult	TGFBR1	190181	AD	1/ 7063	1	1	0	0
	611788		ACTA2	102620	AD	1 / 14125	1	0	0	0
Hypertrophic cardiomyopathy, dilated cardiomyopathy	115197		MYBPC3	600958	AD	1/ 673	1	10	10	0
	192600		MYH7	160760	AD	1/ 1009	4	3	7	0
	601494		TNNT2	191045	AD	1/ 7063	1	1	0	0
	613690	Child/adult	TNNI3	191044	AD	1/ 2825	2	1	2	0
	600858		PRKAG2	602743	AD	1 / 14125	1	0	0	0
	608758		MYL2	160781	AD	1/ 7063	0	2	0	0
	115200		LMNA	150330	AD	1/ 14125	0	0	1	0
Catecholaminergic polymorphic ventricular tachycardia arrhythmogenic right ventricular cardiomyopathy	604772		RYR2	180902	AD	1/ 4708	0	2	1	0
	609040	Child/adult	PKP2	602861	AD	1/ 1413	2	2	6	0
	604400		DSP	125647	AD	1/ 7063	0	0	2	0
	610476		DSC2	125645	AD	1/ 7063	0	1	1	0
Romano-Ward long-QT syndrome types 1, 2,and 3, Brugada syndrome	610193		DSG2	125671	AD	1/ 3531	1	0	2	1
	192500	Child/adult	KCNQ1	607542	AD	1/ 1009	4	3	7	0
	613688		KCNH2	152427	AD	1/ 565	7	7	10	1
	603830/ 601144		SCN5A	600163	AD	1/ 2018	2	2	3	0
Familial hypercholesterolemia	143890	Child/adult	LDLR	606945	AD	1/ 642	2	8	12	0
	603776		APOB	107730	AD	1/ 2825	1	1	2	1
			PCSK9	607786	AD	1/ 2018	0	4	3	0
Ornithine transcarbamylase deficiency	311250	Newborn (male), child (female)	OTC	300461	XL	1/ 3732	1	1	0	0
Malignant hyperthermia susceptibility	145600	Child/adult	RYR1	180901	AD	1/ 642	6	4	11	1
PTEN hamartoma tumor syndrome	153480	Child/adult	PTEN	601728	AD	1/ 14125	0	1	0	0

## Discussion

In this study, we used an unconditioned approach to rank the fetal risk for gene-condition pairs based on individual data from thousands of ES samples complemented by parallel analysis for relevant genes to help inform the development ECS gene-panels and improve clinical strategies for PCS that maximise clinical sensitivity, allow meaningful residual risk calculation and minimising counterproductive effects. Despite using conservative measures, we identified a FDR of 1/337 when combining ES with deep selection of gene-disease pairs and with parallel test for specific relevant genes. This approach has critical advantages compared to carrier rate extrapolation from disease prevalence in the postnatal population because this is usually impacted by ascertain bias, in which only severely affected individuals are identified. Further, the use of individual-level data complemented by parallel testing for specific genes instead of aggregated data from population databases [9], confers significant strength and additional reliability to this study’s findings. These population genetics data can be particularly useful for providers and patients assessing and comparing clinical validity among the heterogeneous PCS strategies and gene-disease panels. Indeed, as shown for couple analysis, most of the available ECS gene-panel designs would have missed a remarkable quote of couple’s at risk for relevant conditions (S4 Table). This observation can be useful for further optimization of ECS sensitivity and harmonization among the PCS offer.

To our knowledge, few ES studies have been conducted on this topic on individuals with no clinical phenotype (except infertility), and those available are based on small sample sizes [9,10] or are especially focused on consanguineous couples [11]. FDR detected in our ES approach was significantly higher than previous pre-selected gene-panel approaches. Exome sequencing aggregated data from gnomAD were recently leveraged by Guo and Gregg to estimate carrier rates across six major ancestries[9]. They showed that screening just the 40 selected genes with carrier rate >1.0% would identify more than 76% of these at-risk couples. Couples at risk were reported in the range of 0.17–2.52% depending on ancestry. However, significant limitations compromising the possibility to accurately estimate FDR in this ES dataset were: the absence of separated tests performed for challenging genes (e.g., deletions in SMN1 causing spinal muscular atrophy); the failure to truly reflect the carrier rates of the individuals who seek carrier screening; and, most importantly, the analysis was limited to a pre-selected list of 415 genes associated with autosomal severe recessive conditions. In the largest ECS study reported to date, Haque and colleagues [2] showed an aggregate FDR of ~1:600 using a pre-selected panel of 94 conditions in a Southern European population. In a more recent study, the same group used twice as many genes (235 genes) than their previous effort, full coverage across coding regions, and panel-wide copy number variation (CNV) calling. Nonetheless, results provided similar clinical sensitivity (4.5% couple at risk) as reported here with half of gene-condition pairs included (4.8% couples at risk)[12]. This is likely explained by the use of an unconditioned approach based on actual ES data that maximise clinical sensitivity of selected gene-disease pairs.

On the contrary, we report a slightly lower carrier burden compared to a recent study evaluating genome sequencing for PCS[10]. That study’s authors analysed a pre-defined set of 728 gene-disorder pairs for carrier screening in 131 women and their partners (n = 71) who were planning a pregnancy, reporting 12 carrier couples. However, this discrepancy is primarily explained by inclusion of gene-disease pairs characterized by adult onset [*SERPINA1* (MIM: 107400); alpha-1 antitrypsin deficiency (MIM: 613490); *HFE* (MIM: 613609) and mild/unpredictable phenotype F5 (MIM: 612309); and factor V Leiden thrombophilia (MIM: 227400)].

In our ECS gene-panel development, the only deviation from scientific recommendations about panel composition involved the expected disease prevalence/carrier rate threshold required for inclusion, which was originally proposed at >1:100[4]. Notably, ES coupled with a deep selection of gene/disorder pairs allowed an increase in testing sensitivity when such lower frequency conditions were considered, highlighting that a significant portion of fetal risk (1 out of 3000 pregnancy) would otherwise be missed.

These results are in line and corroborate previous findings by Ben-Shachar and colleagues[3] obtained from a large data-driven evaluation of ECS clinical detection rate.

Importantly, even with a lower carrier rate, X-linked carriership explained up to 40% of the overall FDR. Specific carrier rate reference values for X-linked conditions are usually neglected from recommendations for ECS gene panel development[13] and data-driven analyses are urgently needed to guide the development of reasonable criteria for X-linked condition for ECS[3]. It should be acknowledged that many X-linked conditions might act in a semi-dominant fashion, such us Ornithine transcarbamylase deficiency (OTC; MIM: 300461) or adrenoleukodystrophy, (ALD; MIM:300100), posing some challenges and subjectivity for testing them in the context of PCS as they can reveal or anticipate a disease trait in carrier women[14]. However, considering the overall contribution to fetal risk and the potential higher severity for hemizygote males, inclusion of severe semi-dominant X-linked conditions for PCS seems reasonable.

At the couple’s risk level, we identified a remarkably higher risk rate (~5%) than expected from aggregate FDR calculations. This discrepancy is partially explained by the random sampling of couples for this analysis and by the imperfect correlation between carrier couples and fetal risk, particularly for *FMR1* pre-mutation carriership. Indeed, most couples’ risk alleles were <70 CGG repeats, with very low risk to full mutation expansion [15]. In this study, the likelihood of *FMR1* expansion to the full mutation size was considered when computing FDR for XFS, while for couple analysis this factor was not accounted for. Nonetheless, this figure was still higher than that extrapolated from FDR data. Considering that the main objective of PCS is to inform couples about their level of risk for recessive diseases before pregnancy, thus improving their reproductive autonomy, couple’s risk data are the best source of information for pre-test reproductive counselling. Therefore, future studies are required to define more accurate estimates of couple’s risk profile based on ECS approach that we propose in this study.

**Fig 4:**
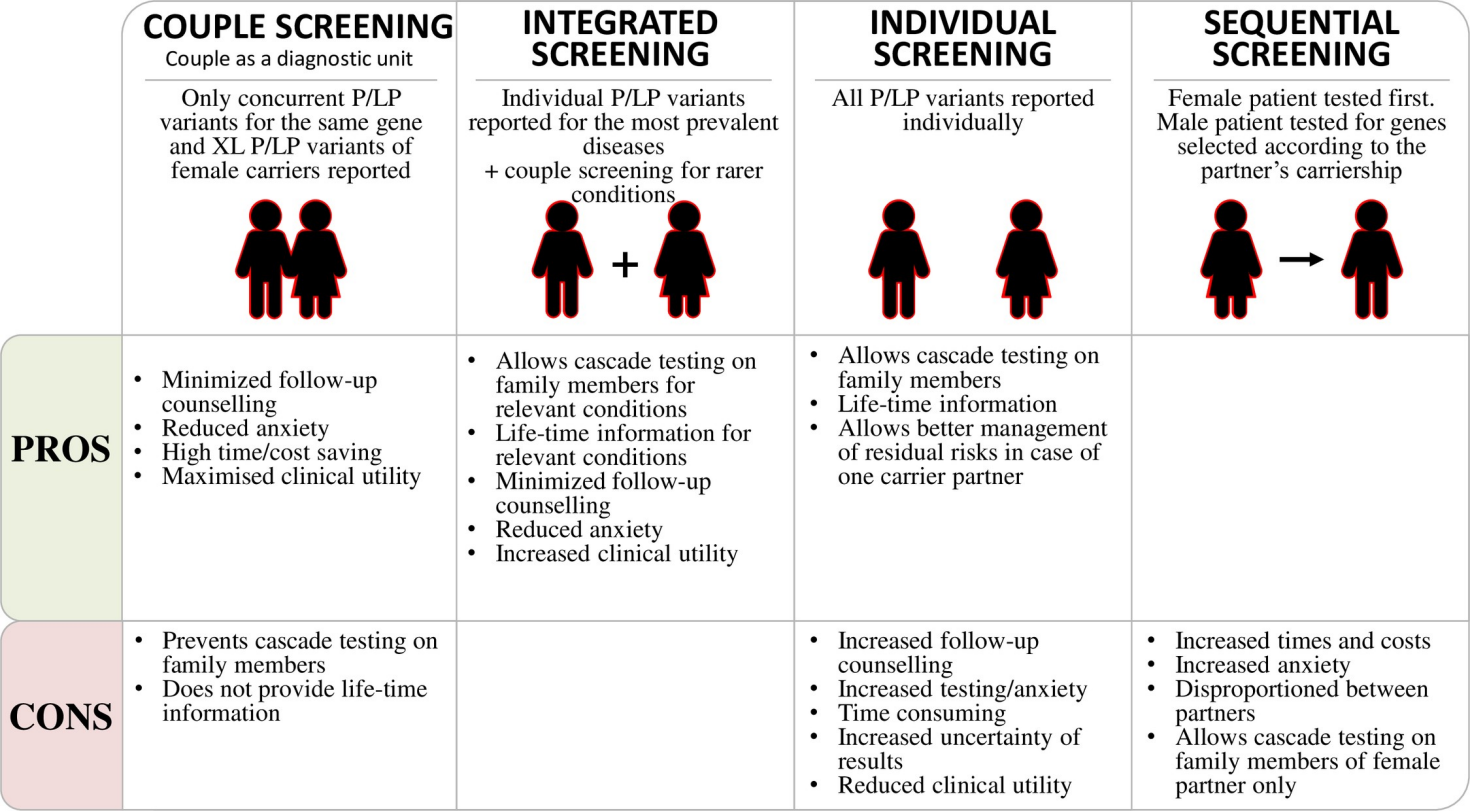
Preconception carrier screening strategies when exome sequencing is used for genetic analysis.

In this subgroup analysis, we also reported an alternative strategy for preconception reproductive risk assessment for very rare conditions that minimize analytical/interpretation/cost burden and yet effectively capture those carrier results likely to have the greatest potential reproductive impact. Indeed, while using genome-wide sequencing for ECS will identify the majority of individuals as a carrier of at least one condition, this raises issues of the practicality of providing every screened individual with information about the condition(s) for which they are carriers. A proposed solution to this concern was “couple screening” [15], where both members of the couple are screened and provided with information about their carrier state only when both members of the couple are carriers of the same autosomal recessive condition or the woman is a carrier of an X-linked condition (Fig 4). If these conditions are not met, they are not provided with their individual carrier status results for autosomal recessive conditions. The advantage of couple screening is that it markedly reduces the time required for genetic counselling for screening programmes. The two major disadvantages of couple screening are that it misses the opportunity for cascade screening and if a couple splits up they may lose clinically relevant and lifetime information about their carrier state. In this study, we have shown the efficacy of an integrated approach that try to maximise the advantages of both couple and individual screening strategies for PCS (Fig 4). After applying our selected gene panel to identify the couple risk, we have expanded the “couple screening” approach to the whole exome content. Two additional couples in our dataset were identified at risk for severe conditions not included in our gene-panel because the absolute carrier rate for them was above our threshold for inclusion, highlighting a potential advantage of using ES-based strategies for PCS to maximise risk detection for meaningful conditions but occurring at very low frequency in the target population. Indeed, both these conditions have not been considered in all commercially available ECS gene-panels assessed here (S4 Table). This integrated approach would not be limited for cascade testing nor for the value of having life-time individual data because for the most prevalent and relevant conditions (ECS gene panel) the carrier status is reported (Fig 4).

### Detection and reporting of medically actionable SF at preconception stage

Our study describes also the carrier burden for SF to prospective parents or gamete donors who were subjected to ES for PCS, of which 2.3% were positive. Recently, multiple studies have reported frequencies of SF ranging 1%–9% in various populations[16–23]. It is thus expected, as observed here, that clinically significant variants with reduced penetrance and adult-onset conditions are detected with considerable frequency. While a majority of individuals are generally willing to receive identified actionable SF[24] and disclosure of positive results shows little to no adverse impact on participants and adds only modestly to near-term health-care costs (Hart MR 2019), the impact of reporting SF in IVF patients/gamete donors has not yet been sufficiently addressed. In 2014, a European Society of Human Reproduction and Embryology (ESHRE) task force[25] supported a broader view on preimplantation genetic testing for monogenic disease (PGT-M), including the health of third generations, which should be considered in light of recent developments in exome/genome sequencing-based PCS. Indeed, the recent increase in cancer predisposition genetic assessment in the general population has been followed by increased demand for PGT-M in Europe for such conditions. In the most recent ESHRE data from 2016[26], breast cancer ranked second among all conditions tested with PGT in Europe, exceeding CF and the most common conditions. In this context, SF reporting might be perceived in line with the commitment to enhance patient reproductive autonomy, as carrier couples for oncogenic conditions elect PGT-M to prevent risk of transmission.

However, the utility of returning SF to facilitate preventive screening/actions needs to be further addressed and balanced with an over-uptake of preimplantation/prenatal diagnosis. Also, the possibility to access PGT-M/prenatal diagnosis programs in each specific clinical and social setting[5,27] must be carefully evaluated. In this light, a reasonable approach to compile panels for SF should consider eligibility of the condition for PGT-M/prenatal testing. Alternatively, limitations for reproductive genetic testing should be made clear to prospective parents when opting for SF data reporting.

For gamete donors, the situation is even less defined. On one hand, donors might consider SF reporting an inherent health benefit and perceive this as additional "compensation" for their donation. On the other hand, the carrier burden for SF might reduce gamete availability and increase costs of donor programs. Future studies are required to investigate the clinical utility and impact of returning SF in the reproductive medicine practice.

### Limitations

The lack of ethnic diversity in our dataset represents some limitations to our wider conclusions. However, disease-specific frequencies provided in the Supplemental Data should allow comparisons with ES data collected in other preconception populations with different ethnicities. Of note, for a broadly generalizability of our findings, conditions known to be highly relevant in specific ancestries, such as Ashkenazi, need to be considered for inclusion when aiming at developing universal ECS panels. Moreover, the high carrier rate observed for Fragile X Syndrome (1/148) can be partially explained by ascertainment bias due to the infertility condition of many women included in the study.

On the analytical side, the current ES protocol lacks chromosome copy number (CNVs) and non-coding pathogenic variant analysis. Recent studies pointed out a considerable contribution of pathogenic CNVs in carrier risk assessment[10,12,28]. In time, improved ES data analysis pipelines and increased use of genome sequencing will further increase completeness of the data[29,30].

Moreover, variant classification remains an important constraint in clinical exome/genome sequencing. Notably, ACMG criteria for variant classification have been divergently interpreted among laboratories[31]. We selected pathogenic variants based on the last release of public databases (ClinVar[32]) and intra-laboratory predictions by the nature of variant frequency and homozygosity in healthy individuals, which itself is an imperfect methodology. Further, we did not select non-annotated (likely) pathogenic null variants in our strategy to provide a conservative approach to reproductive risk estimates. Obtaining more experience in a translational genomic research setting with the nature of variants for both common and rare gene-condition pairs will improve pathogenicity prediction in clinical practice[33]. At present, developing and constantly updating a standardized variant/gene list for PCS where accurate gene-level clinical sensitivity and residual risks are available can significantly mitigate these clinical challenges.

## Conclusions

Taken together, this study on large translational genomic research data will facilitate development of more effective ECS gene panels and PCS strategies that maximize clinical sensitivity with minimal counterproductive effects. The possibility of effectively defining the couples’ genetic risk by current PCS strategies is crucial for disease prevention in human pregnancies and for improving couples’ reproductive autonomy. It is also possible to anticipate that in consideration of the constant evolution and uptake of genome sequencing in different fields of medicine, the scope of preconception genetic investigation will likely expand significantly in the coming years[30,34]. For instance, in the specific reproductive medicine context, exome/genome sequencing at preconception will help explain and better manage some idiopathic cases of infertility or anticipate specific phenotype and IVF treatment outcomes[35,36]. However, while this study addresses one crucial component to determine criteria for an ECS gene-panel development and implementation, decisions and recommendations about how to implement ES-based PCS will require further information. Examining medical, educational, behavioural, and economic outcomes of ES-based PCS implementation to healthy individuals is a matter that requires further research, which is ongoing[37]. For instance, incorporation of mild conditions or adult-onset conditions involves values and preferences that will not be solely driven by considerations addressed by this manuscript. Also, the best framework to educate healthcare providers and patients undergoing PCS based on genome-wide sequencing is still a poorly developed field in many countries, but it is a matter of particular relevance that needs further considerations to facilitate equity to information access and informed decision-making.

## Methods

### Ethics statement

The study protocol was approved by the Ethics Committee of the Hospital Clinic Universitari de Valencia, Spain (number 2018/279). Consent was not obtained because the data were analyzed anonymously.

### Design and data set

This study included anonymised ES results of 14,125 individuals undergoing PCS at Igenomix-affiliated clinics during September 2015–March 2018. Among these, 8,280 individuals were from couples undergoing IVF (6,334 males and 1,946 females), and 5,845 were gamete donors (327 males and 5,518 females). Prior to anonymization, diagnostic data from ES and separate tests for specific genes were used to calculate each patient’s carrier status based on a pre-defined list of target variants/genes[38]. Then, exome sequencing and separate tests data were anonymised and analysed to define fetal risk at both gene-disease and aggregated levels. To minimize bias in disease frequency calculations, genetic data were used only if the patient reported no remarkable personal or family history of carrier status or genetic disease following a specific counselling session with the reproductive physician. Family history analysis was reported on PCS requisition forms and the selection of samples to be included in the study was based on the absence of relevant genetic data reported by the doctor. Information about a couple's risk profile was possible from 776 couples (where both members underwent ECS by ES and separate tests) due to common use of the "one-member screening strategy" in PCS for IVF couples (only one member of the couple performs the ECS and residual risk are evaluated based on population carrier frequency data), while the remaining 40% of samples were from donors. Nonetheless, for the preliminary phase of this study, the use of actual couples' data was not strictly necessary because gene-level and aggregated fetal disease risk was calculated from carrier rate values determined from the large sample size. After the gene-panel development, couple’s risk was evaluated on actual data from 776 couples.

As the offer of testing was at the discretion of individual IVF clinics, we could not collect complete medical histories, medical records, prior and after testing, from these subjects. The majority of participants was of European descent, particularly Southern European.

### Sequencing, variant filtering/annotation, and separate tests

Massively parallel sequencing was performed on the NextSeq 500 platform (Illumina). Briefly, sequence-enrichment DNA probes were commercially obtained using the Trusight One system (Illumina) and included all coding exons with flanking 10-bp intronic sequences of the targeted 4,813 genes. Each DNA sample was indexed during library preparation, and 24 samples were sequenced (PE150) on each flowcell of the NextSeq 500 platform. Sequence data analysis was performed using the Illumina bioinformatics analysis pipeline (bcltofastq). Briefly, the pipeline was used for base-calling and to separate each barcoded data set. Illumina paired-end reads were aligned to the reference human genome build hg19 using bwa-mem[39]. Standard bioinformatics tools were used for PCR duplicate subtraction, mapped reads filtering, and sorting/indexing mapping files[40,41]. Raw variants were called using Freebayes, and functional and database annotation was done with SnpEff [42]. ClinVar database (release 20180225)[32] was used to clinically interpret variants. Sequences with less than 10X coverage and SNVs with <35% heterozygous ratio and having a base call quality scores ≤Q20 were not considered for the analysis[43–45]. Considering previous validation performed on the NGS sequencing assay, the use of stringent quality metric threshold for variant calling and the translational research setting of this analysis, novel SNVs were not confirmed by orthologous methods.

Current ES technologies are incapable of detecting all variants relevant for PCS, such as those causing triplet repeat disorders [e.g., FXS (MIM: 300624)] and genomic regions with high homology (pseudogenes). Due to this inherent limitation, multiple methodologies were used to detect the full range of pathogenic variant classes in well-characterized genes. Accordingly, the PCS strategy used in most cases included ES and separate tests for *HBA* (MIM: 141800), *SMN1* (MIM: 600354), and *GBA* (MIM: 606463) for all patients, and *DMD* (MIM: 300377) and *FMR1* CGG pre-mutation sizing for females only, as previously described[38]. Separate test data are available for most PCS cases in this dataset.

### Data analysis and gene-disease pair exclusion

ES and separate gene test data were elaborated in a stepwise approach to define carrier rate and gene-disease level and aggregated fetal risk (Fig 1; S1 and S2 Tables). Single nucleotide variants (SNVs) were individually assessed to remove non-pathogenic variants, variants of unknown significance (VUS), and false positive calls. Homozygote variants detected in healthy subjects and heterozygote variants with an allele frequency higher than a single nucleotide polymorphism (SNP) [minor allele frequency (MAF) > 1%] were excluded (Fig 1). Only pathogenic (P) and likely pathogenic (LP) variants according to the last ClinVAr[32] classification were included (ClinVar: 20190325). Although our study began prior to publication of the formal classification system proposed by ACMG[46], our interpretation criteria are conceptually similar. As a general assumption and trade-off between accurate representation and interpretative process, variants with known low penetrance or mild phenotype were excluded from analysis, while all included variants were treated as having an equal phenotypic impact.

Subsequent data analysis involved step-wise exclusion of gene-disease pairs depending on the following main criteria: carrier frequencies, inheritance pattern, age of onset, penetrance, and strength of gene-disease association. Although there is no ideal and common threshold to determine which conditions to include in an ECS panel in relation to carrier rate and disease risk, a disease prevalence of 1 in 1 million [fetal disease risk (FDR) of 1*10^−6^; carrier frequency >1 in 500 for autosomal-recessive (AR) conditions] was used as a threshold for conditions in this study. This threshold was set to provide a meaningful representation of gene-disease-specific and aggregate FDRs for more conditions than previously possible in large studies using a preselected panel of conditions[2] and also considering available sample size. Next, gene-disease pairs associated with recessive inheritance were excluded if classified as low/moderately penetrant or late-onset using previously described criteria[47]. Finally, gene-disease pairs without records or with low/moderate evidence of gene-disease association according to the Clinical Genome Resource’s[48] framework were excluded (S2 Table).

Condition of severity was ranked on an ordinal scale as previously described (profound, 4; severe, 3; moderate, 2; and mild, 1)[49]. All gene-condition pairs excluded in this stepwise approach are reported in S2 Table, along with reasons for exclusion.

### Fetal risk calculation and outcome measures

To account for the impact of different inheritance patterns on fetal risk, carrier rate for each gene was used to compute FDR, as previously described by Haque and colleagues[2]. This outcome measure statistically quantifies the rate of affected conceptuses based on carrier frequency data, accounting for the specific inheritance pattern and using simulated parental populations. To account for the specific inheritance pattern and molecular basis of some diseases, further elaboration of carrier rate was performed for separately tested conditions. In particular, for FXS, fetal risk is not easily inferred from carrier frequency and requires a risk model that considers the probability of repeat expansion as a function of maternal CGG repeat number in *FMR1*[50]. For HBA, fetal risk was computed considering the likelihood of a carrier of –α3.7 variant to match with a carrier of–MED or–SEA variants.

Gene-specific and aggregate FDR were calculated for all gene-pair conditions, combining results from ES and all complementary tests (Fig 1).

### Assessment of secondary findings from exome data

Participants’ exome variants were reviewed for the 59 genes of interest (ACMG SF v2.0)[8] for each variant listed as P/LP according to Varsome’s ACMG classification[51]. However, “disease-causing” variants were assumed to be benign for rare autosomal-dominant (AD) disorders when MAF > 0.005, as they were too common to be highly penetrant pathogenic variants given the disease frequency. Of note, the original and updated ACMG SF v2.0 recommendations use terms “known pathogenic” and “expected pathogenic” when considering which variants to return. In this analysis, we disclosed variants as P and LP, consistent with ACMG/AMP recommendations for interpreting pathogenicity of sequencing variants[46].

### Statistical analysis

Continuous variables are shown as mean ± SD and range. T-tests or Mann-Whitney U tests were conducted to assess statistical significance of differences for continuous variables. Categorical variables are shown as percentages with 95% confidence intervals (95% CI). Fisher’s exact test was conducted to assess statistical differences between groups of subjects undergoing ES according to their gender and/or indication to PCS (IVF couples/gamete donors). *P* < 0.05 was considered statistically significant.

## Supporting information

S1 Table Pathogenic and likely pathogenic variants used to compute carrier rates and FDR for each specific gene-condition pair including separated tests for challenging genes. (XLSX)

S2 Table Step-wise exclusion of gene-disease pairs depending on the main criteria of carrier frequencies, inheritance pattern, age of onset, penetrance, and strength of gene-disease association. The final list of curated gene-condition pairs included in the final panel is reported in the “curated gene” sheet with diseases characteristics, observed carrier rate and modelled fetal disease risk.
(XLSX)

S3 Table Pathogenic and likely pathogenic variants detected for secondary findings genes in the studied population. (XLSX)

S4 Table Analysis of gene-disease coverage for the standard expanded carrier screening gene-panels available on the market in relation to the couples at risk detected in this dataset by exome sequencing and parallel gene testing for selected conditions. Few frequent severe and moderate conditions are consistently missing by all assessed expanded carrier screening gene-panels.
(XLSX)
